# Editorial: Reviews in antibiotic resistance and new antimicrobial drugs

**DOI:** 10.3389/fcimb.2024.1434140

**Published:** 2024-06-04

**Authors:** Sanket Kaushik

**Affiliations:** Amity Institute of Biotechnology, Amity University Rajasthan, Jaipur, Rajasthan, India

**Keywords:** multi drug resistance (MDR), biofilm, antimicrobial resistance, ESKAPE, bacterial infections

At the time of their discovery, antibiotics were considered a wonder drug for the treatment of bacterial infections, but the way microorganisms have involved several robust mechanisms to encounter the antibiotics has given rise to multi-drug-resistant (MDR) and extensively drug-resistant (XDR) strains (Eichenberger and Thaden, 2019; Terreni et al., 2021; Yadav et al., 2022). There are several ways by which the pathogen acquires resistance to antibiotics, including genetic variation in the genome of the pathogen, unselective usage of antibiotics, the development of biofilms, etc (Santos-Lopez et al., 2019; Singh et al., 2019). Since it has become difficult to treat such infections, it is essential to understand the mechanism of the development of anti-microbial resistance (AMR) so that strategies to prevent such infections can be developed (Lomazzi et al., 2019; Hu et al., 2020; Moo et al., 2020).

The goal of this Research Topic was to highlight recent advances in the field of antibiotic resistance while emphasising important directions and new possibilities for future inquiries. We anticipate the research being presented here will ignite conversations within the community about novel antimicrobial medications and antibiotic resistance, which will lead to the application of best practices in clinical, public health, and policy settings.

Overall, four research articles and six review articles were published in this Research Topic. A research article published by Wang et al. reported the epidemiological studies of nontuberculous mycobacteria (NTM) in tuberculosis suspects in the southwest of China from 2017 to 2022. In this study, the main NTM isolates, MAC and *M. chelonae/M. abscessus*, were identified, and it was observed that the isolation rate of NTM in southwest China has shown an increasing trend in the last few years. The infected cases were elderly patients, individuals with compromised immune systems who had an HIV infection. On evaluation, it was observed that antibiotics like amikacin, moxifloxacin, clarithromycin, and linezolid demonstrated effective antibacterial activity against slow-growing mycobacteria, whereas linezolid and amikacin exhibited relatively better antibacterial activity against rapid-growing mycobacteria. Another research article published by Shi et al. studied the prevalence and resistance characteristics of MDR *Streptococcus pneumoniae* isolated from the respiratory tracts of hospitalised children in Shenzhen, China. It was observed that the non-vaccine serotype strains accounted for 46.6% of all the pneumococcal isolated strains. The multidrug resistance rates (MDR) of vaccine serotypes were 19F (99.36%), 19A (100%), 23F (98.08%), 6B (100%), and 6C (100%), and the MDR of non-vaccine serotypes were 15B (100.00%), 6E (100%), 15C (100%), 34 (95.24%), and 23A (98.31%), respectively. Data indicated that there has been a notable rise and spread of multidrug-resistant non-vaccine serotypes among children. Another research paper is entitled “*PqsA* mutation-mediated enhancement of phage-mediated combat against *Pseudomonas aeruginosa.”* In this study, the *PqsA* gene was highlighted as a potential drug target to enhance phage therapy, as the deletion of the *pqsA* gene could significantly promote the lysis of phages on *Pseudomonas aeruginosa. A research* article by Janc et al. highlighted the impact of *Klebsiella pneumoniae* NDM (New Delhi metallo-B-lactamase) infection and other bacterial infections on mortality in patients treated in ICUs due to COVID-19. It was reported that in patients treated for SARS-CoV-2 infection, acquiring a bacterial infection due to prolonged hospitalisation associated with the treatment of COVID-19 did not elevate mortality rates. The data also suggested that in severe COVID-19 patients who survived beyond the first week of hospitalisation, bacterial infections, particularly *Klebsiella pneumoniae* NDM, do not significantly impact mortality.

A review article published by Ari et al. highlighted the properties and potential of nitrofurantoin (NF) in the treatment of urinary tract infections (UTI). The author has studied the detailed pharmacokinetic and pharmacodynamic properties along with the antibacterial activity and mechanism of action of the drug and concluded that NF can be considered the most effective drug in the treatment of acute urinary infection, but due to the long-term side effects of this drug, especially in elderly patients, it is essential to introduce some criteria for prescribing NF in cases of chronic UTI. Another review article by Wang et al. highlighted antimicrobial resistance and its mechanism of epigenetic regulation. The author has extensively focused on the effects of DNA modification, histone modification, rRNA methylation, and the regulation of non-coding RNA expression on antimicrobial resistance. Bacterial epigenetics, modifications of DNA and rRNA, ncRNAs, as well as nucleoid-associated proteins, have been shown to regulate the development of AMR. Another review paper entitled “Application of the CRISPR-Cas system in the diagnosis and therapy of ESKAPE infections” elaborated on the applications of the CRISPR-Cas system for the study of ESKAPE pathogens. The author highlighted that although currently no direct CRISPR-based anti-infective treatment methods are available, the CRISPR-Cas method can be a promising alternative to treatment because of its specificity.

A review paper by Schami et al. focused on the cell envelope profiles of drug-resistant strains of *Mycobacterium tuberculosis* and their interactions with the host. The composition and complexity of the cell envelope were discussed, along with its importance as a drug target for the development of anti-bacterial drugs. The author has also described the current knowledge regarding the influence of drug resistance on infection outcomes. Another review paper by Pai et al. has highlighted the need to design novel compounds for the eradication of infections caused by MDR bacteria. Different strategies by which bacteria gain resistance to several antibiotics have been discussed, along with the pathways that can be targeted for development by antimicrobial drugs with better potency. One more review paper by Ramirez et al. emphasised the application of anti-microbial peptides in livestock farming and how they can mitigate the impact of this practice within the One Health framework. Despite several challenges, the pace at which bacteria adapt to these peptides is very slow as compared to other methods. Therefore, AMPs offer a potential solution to the scarcity of effective antibiotics against MDR bacteria.

## Author contributions

SK: Writing – original draft, Writing – review & editing.
